# A Multicomponent Neuronal Response Encodes the Larval Decision to Pupariate upon Amino Acid Starvation

**DOI:** 10.1523/JNEUROSCI.1163-18.2018

**Published:** 2018-11-21

**Authors:** Siddharth Jayakumar, Shlesha Richhariya, Bipan Kumar Deb, Gaiti Hasan

**Affiliations:** National Centre for Biological Sciences, TIFR, Bangalore 560065

**Keywords:** *Drosophila*, glutamatergic neurons, nutrient-sensing, SOCE

## Abstract

Organisms need to coordinate growth with development, particularly in the context of nutrient availability. Thus, multiple ways have evolved to survive extrinsic nutrient deprivation during development. In *Drosophila*, growth occurs during larval development. Larvae are thus critically dependent on nutritional inputs; but after critical weight, they pupariate even when starved. How nutrient availability is coupled to the internal metabolic state for the decision to pupariate needs better understanding. We had earlier identified glutamatergic interneurons in the ventral ganglion that regulate pupariation on a protein-deficient diet. Here we report that *Drosophila* third instar larvae (either sex) sense arginine to evaluate their nutrient environment using an amino acid transporter Slimfast. The glutamatergic interneurons integrate external protein availability with internal metabolic state through neuropeptide signals. IP_3_-mediated calcium release and store-operated calcium entry are essential in these glutamatergic neurons for such integration and alter neuronal function by reducing the expression of multiple ion channels.

**SIGNIFICANCE STATEMENT** Coordinating growth with development, in the context of nutrient availability is a challenge for all organisms in nature. After attainment of “critical weight,” insect larvae can pupariate, even in the absence of nutrition. Mechanism(s) that stimulate appropriate cellular responses and allow normal development on a nutritionally deficient diet remain to be understood. Here, we demonstrate that nutritional deprivation, in postcritical weight larvae, is sensed by special sensory neurons through an amino acid transporter that detects loss of environmental arginine. This information is integrated by glutamatergic interneurons with the internal metabolic state through neuropeptide signals. These glutamatergic interneurons require calcium-signaling-regulated expression of a host of neuronal channels to generate complex calcium signals essential for pupariation on a protein-deficient diet.

## Introduction

The coordination of organismal growth with development requires monitoring of nutrient availability and its integration with the internal metabolic state (Boulan et al., 2015). How nutrients inform developmental decisions, however, remains poorly understood. An example of a nutrient dependent developmental decision is pupation in insects (Nijhout and Williams, 1974). In *Drosophila*, third instar larvae feed voraciously and undergo an important developmental checkpoint based on their nutritional status, which is the achievement of critical weight, when larvae can pupariate even upon subsequent starvation (Rewitz et al., 2013). Changes in neuroendocrine signals that might encode the decision to pupariate postcritical weight need elucidation (Nijhout and Williams, 1974; Mirth et al., 2005; Rewitz et al., 2013).

Amino acid sensors, such as GCN2 (General Control Nonderepressible 2) and mTOR, have been thought to encode responses to nutrient deprivation (Hao et al., 2005; Bjordal et al., 2014; Goberdhan et al., 2016), although a GCN2-independent mechanism was described previously (Leib and Knight, 2015). Interestingly, specific but different amino acids elicit differing responses upon encountering a nutrient-poor diet in *Drosophila* (Bjordal et al., 2014; Corrales-Carvajal et al., 2016; Croset et al., 2016). Moreover, loss of amino acid alters the internal metabolic state and affects systemic behavior (Ribeiro and Dickson, 2010; Fontana and Partridge, 2015; Corrales-Carvajal et al., 2016). In a recent study, we demonstrated that the ability to pupariate postcritical weight in the absence of EAAs requires cholinergic stimulation of the mAChR present on a set of glutamatergic interneurons in the ventral ganglion (VG) (Jayakumar et al., 2016). These glutamatergic neurons lie at the heart of a neural circuit required for the decision to pupariate under protein starvation and depend on intracellular calcium signaling. The nature of the sensory information received and its integration with stage-specific internal metabolic state(s) warranted further investigation.

The mechanism(s) by which intracellular calcium signaling in neurons modulates cell function is only beginning to be understood (Hartmann et al., 2014; Richhariya et al., 2017; de Juan-Sanz et al., 2017). Similar to what is known from immune cells (Feske, 2007), intracellular calcium signaling through the IP_3_R and Store-operated calcium entry (SOCE) has recently been implicated in regulating gene expression in excitable cells (Somasundaram et al., 2014; Richhariya et al., 2017).

We report here that loss of environmental amino acids, especially arginine, elicits complex calcium transients in a population of glutamatergic neurons. These transients are shaped both by sensory inputs and neuropeptidergic modulation. Gene expression analysis of the glutamatergic interneurons supports SOCE-regulated gene expression as a driver of neuronal plasticity required for handling nutrient stress during development.

## Materials and Methods

### 

#### 

##### Fly stocks and rearing.

*Drosophila* strains were grown on cornmeal medium supplemented with yeast (Normal Diet [ND]) as described by Subramanian et al. (2013) at 25°C unless otherwise noted. The protein-deprived diet (PDD) contained 100 mm sucrose with 1% agar. For the single amino acid rescues, corresponding amino acids at 1× concentration of the commercially available EAA Mixture (Invitrogen) were added to PDD. Single amino acids were obtained from Sigma-Aldrich and used at the following concentrations (in mm): l-arginine 2.995, l-cystine 0.5, l-histidine 1, l-isoleucine 2, l-leucine 2, l-lysine 1.98, l-methionine 0.505, l-phenylalanine 1, l-threonine 2, l-tryptophan 0.25, l-tyrosine 0.99, and l-valine 2. For optogenetic experiments, egg laying was performed in ND supplemented with 200 μm all-trans-retinal, and larvae were transferred at 84 ± 4 h onto the ND with 400 μm all-trans-retinal. A table of all stocks used is appended as Table 1. The *itpr IR* was used with *UAS-dicer2* in all experiments.

**Table 1.** List of fly stocks

Fly line	Description	Source
*VGN6341-GAL4*	Subset glutamatergic driver	Syed et al., 2015
*ppk-GAL4*	Class IV multidendritic driver (very weak expression in Class III)	BL32078; Zhong et al., 2010
*OK6-GAL4*	Motoneuron driver	Aberle et al., 2002
*Dilp2-GAL4*	Expresses GAL4 in insulin-producing cells from the mNSCs (Dilp2 driver)	Rulifson et al., 2002
*PTTH-GAL4*	PTTH driver	McBrayer et al., 2007
*FMRFa-GAL4*	FMRFa driver	Park et al., 2008; gift from Paul Taghert
*Cg-GAL4*	Fat-body driver	BL7011
*UAS-ANF::GFP*	Expresses ANF::GFP	BL 7001; Rao et al., 2001
*UAS-Shi^ts^*	Inhibits vesicle recycling	Kitamoto, 2001
*UAS-ChR2.XXL*	Channelrhodopsin variant	Dawydow et al., 2014; gift from Christian Wegener
*UAS-itpr IR; UAS-dicer*	RNAi line for *itpr* combined with dicer	1063-R2 from NIG
*UAS-dStim IR; UAS-dicer*	RNAi line for *dStim* combined with dicer	V47073/GD from VDRC
*UAS-dOrai IR; UAS-dicer*	RNAi line for *dOrai* combined with dicer	12221/GD from VDRC
*UAS-mAChR60C IR*	RNAi line for mAChR	VDRC 101407 CG4356; Agrawal et al., 2013
*UAS-Kir2.1*	Inhibitor of neuronal activity (inward rectifying potassium channel)	Baines et al., 2001
*UAS-eGFP*	Cytosolic GFP	Gift from Michael Rosbash
*UAS-FMRFaR IR*	RNAi for FMRFaR	CG2114-R1 from NIG
*UAS-CCHa2R IR*	RNAi for CCHa2R	CG14593, 1658 from VDRC
*UAS-AstAR IR*	RNAi for AstA R	CG10001, 108648 from VDRC
*UAS-slif IR*	RNAi for slif	CG11128 v45589 from VDRC
*UAS-CCHa2 IR*	RNAi for CCHa2	BL57183
*UAS-GCaMP6m*	Genetically encoded calcium indicator	BL42748
*UAS-jRCaMP1b*	Red-shifted calcium indicator	BL63793; Dana et al., 2016
*UAS-PLTXII*	Toxin against presynaptic insect Ca^2+^ channels	Wu et al., 2008
*UAS-*κ*-ACTX-Hv1c*	Toxin against K^+^ channels	Wu et al., 2008
*UAS-*δ*-ACTX-Hv1a*	Toxin against Na^+^ channels	Wu et al., 2008
*UAS-CsChrimson*	Red-shifted optogenetic activator	BL 55135; Klapoetke et al., 2014
*UAS-eNpHR2*	Optogenetic inhibitor (chloride channel)	Gift from Leslie Griffith; Berni et al., 2012
*dimm-LexA(C929)*	LexA under *dimm* promoter	Jayakumar et al., 2016
*LexAop-ANF::GFP*	Expresses ANF::GFP under LexAop	Jayakumar et al., 2016
*LexAop-Shi^ts^*	Shi^ts^ under LexAop	BL44276; T. W. Chen et al., 2013
*ppk-QF*	Class IV multidendritic driver (very weak expression in Class III)	BL36348; Petersen and Stowers, 2011
*QUAS-eNpHR3*	Optogenetic inhibitor (chloride channel) under QUAS	BL 36355; Petersen and Stowers, 2011

##### Pupariation assay.

Larvae at 84 ± 4 h after egg laying (AEL) of either sex were transferred to desired media in batches of 25 and were scored for pupariation after 10 d. At least six independent batches were performed for each genotype on each media. These are reported as percentage pupariation. For rate of pupariation, genotypes were monitored every 12 h after transfer of larvae.

##### Live imaging from larval CNSs.

Larval CNS were dissected in hemolymph-like saline (HL3) (70 mm NaCl, 5 mm KCl, 20 mm MgCl_2_, 10 mm NaHCO_3_, 5 mm trehalose, 115 mm sucrose, 5 mm HEPES, 1.5 mm Ca^2+^, pH 7.2), embedded in 0.2% low-melt agarose (Invitrogen), and bathed in HL3. GCaMP6m was used as the genetically encoded calcium sensor. ANF::GFP was expressed genetically to quantify vesicular release. For creating the effect of an acute loss of amino acid levels, we incubated the *ex vivo* preparations in either 0.5× EAA, obtained from a 50× EAA mixture lacking glutamine (Thermo Fisher Scientific) or 0.3 mm arginine (Sigma-Aldrich), dissolved in HL3. At the point of withdrawal, the amino acid levels were diluted 10-fold using more HL3 thus creating the effect of amino acids withdrawal. Mock withdrawals were performed by increasing the volume without changing the amino acid concentrations. For recording from Class IV multidendritic neurons on the cuticle, semi-intact preparations were used where the CNS along with the anterior half of the larva was embedded in 0.2% low-melt agarose.

Images were taken as a time series on an *xy* plane across 6 *z* planes at an interval of 4 s using a 10× objective with an NA of 0.4 and an optical zoom of 4 on an SP5 inverted confocal microscope with a resonant scanner at 8 kHz (Leica Microsystems). A *z* project across time was then obtained as a time series. This time series across depth was then used for further analysis. For optogenetic inhibition experiments, a green 561 nm laser line was driven simultaneously with image acquisition scans using the 488 nm laser line. Conversely, for activation experiments, a 488 nm laser was driven simultaneously while acquiring image scans with the 561 nm laser. All live imaging experiments were performed with at least five independent CNS preparations, and the exact number of cells for each experiment is indicated in the figures.

Raw images were extracted using Image J1.48 and ROIs selected using the Time Series Analyzer plugin (Balaji, https://imagej.nih.gov/ij/plugins/time-series.html). ΔF/F was calculated using the formula ΔF/F = (F_t_ − F_0_)/F_0_, where F_t_ is the fluorescence at time t and F_0_ is baseline fluorescence corresponding to the average fluorescence over the first 10 frames. Any cell in which the GCaMP signal rose above an arbitrary value of ΔF/F = 1.5 at any point after withdrawal or stimulation was classified as a responder. Percent responders were calculated as follows: (number of responder cells/total number of cells) × 100. ROI heatmaps were mapped on to segments T3-A5 of the CNS, and then percentage responders in each segment were plotted using Matrix2png (Pavlidis and Noble, 2003). Area under the curve was calculated from the point of stimulation until 600 s using Excel (Microsoft) and plotted using BoxPlotR (Spitzer et al., 2014).

For measuring peptide release, the decrease in intracellular fluorescence was measured and quantified as ΔF/F. Release was calculated by (F_0_ − F_t_)/F_0_, where F_t_ is the fluorescence at time t and F_0_ is baseline fluorescence corresponding to the average fluorescence over the first 10 frames. Area under the curve was calculated from point of withdrawal to 600 s using Excel (Microsoft), and box plots were plotted using BoxPlotR (Spitzer et al., 2014). Peptide sequences used were FMRFa-DPKQDFMRFa (NeoBioLab), CCHamide-2-GCQAYGHVCYGGH-NH2, and Allatostatin-SRPYSFGL-NH2 (LifeTein).

##### Immunohistochemistry.

To visualize CaLexA-GFP, larvae were either exposed to ND or PDD for 12 h. The CNS were then dissected in cold PBS, fixed with 4% PFA, washed with 0.2% PTX, blocked and incubated overnight in primary rabbit anti-GFP antibody (1:10,000; A6455, Invitrogen, RRID:AB_221570). They were then washed and incubated with an anti-rabbit AlexaFluor-488 (#A1108, Invitrogen, RRID:AB_143165) and mounted. Confocal images were obtained on the Confocal FV1000 microscope (Olympus) with a 20×, 0.7 NA objective. Images were visualized using either the FV10-ASW 4.0 viewer (Olympus) or Fiji (RRID:SCR_002285) (Schindelin et al., 2012).

##### RNA-seq from larval CNSs.

RNA was isolated from ∼15 larval CNS of 84(±1) h AEL larvae of both control (*UAS-itpr IR/+; UAS-dicer2*/+) and *itpr* KD (*VGN6341GAL4*>*UAS-itpr IR; UAS-dicer2*) genotypes using Trizol (Thermo Fisher Scientific) following the manufacturer's protocol. Libraries with ∼500 ng total RNA per sample were prepared as described previously (Richhariya et al., 2017). Libraries were run on a Hiseq2500 platform at AgriGenome labs. Biological triplicates were performed for the control and duplicates were performed for *itpr* KD.

Per sample, 45–73 million reads were obtained after sequencing. Sequencing reads were aligned to the dm6 release of the *Drosophila* genome using TopHat (Trapnell et al., 2009) and >97% mapping was obtained for all samples. PCR duplicates were identified and removed using Samtools (Li et al., 2009) (http://samtools.sourceforge.net); 18%–23% reads were unique and correspond to 10–14 million reads. These unique reads were used for further analysis. Differential expression analysis was performed using three independent methods: CuffDiff2 (Trapnell et al., 2013), DESeq (Anders and Huber, 2010), and edgeR (Robinson et al., 2010). Alignment files obtained from TopHat were used for differential analysis with CuffDiff2. Read counts were also obtained by GenomicRanges (Lawrence et al., 2013), which were then used to estimate differential expression by DESeq and edgeR. A fold change cutoff of a minimum 25% change was used. Genes with nonzero values in both conditions were considered. Significance cutoff was set at *q* value of <0.05 for CuffDiff2 and FDR-corrected *p* value of <0.05 for DESeq and edgeR. Heat maps were generated using Matrix2png (Pavlidis and Noble, 2003). Comparison of gene lists and generation of Venn diagrams was performed using Whitehead BaRC public tools (http://jura.wi.mit.edu/bioc/tools/). Gene ontology analysis for molecular function was performed using two platforms: GOrilla (Eden et al., 2009) and Panther GO-slim (Mi et al., 2017). In both methods, 287 downregulated genes were used as the target set and all genes in *Drosophila* were used as background.

##### Cell sorting.

FACS was used to sort cells of interest from larval CNS, where neurons of interest were genetically labeled by GFP using the GAL4/UAS system. Control (*VGN6341-GAL4*>*UAS-eGFP*) and *dStim* KD (*VGN6341-GAL4*>*eGFP; UAS-dStim IR; UAS-dcr2*) CNSs were dissected in Schneider's medium (Thermo Fisher Scientific). Approximately 20 CNSs were pooled per sample. These CNSs were treated with an enzyme solution (0.75 μg/μl collagenase and 0.4 μg/μl dispase in Schneider's medium) at room temperature for 30 min. They were then washed and resuspended in cold Schneider's medium and gently triturated several times using a pipette tip to obtain a single-cell suspension. This suspension was then passed through a 40 μm mesh filter to remove clumps and kept on ice until sorting (less than an hour). Flow cytometry was performed on a FACS Aria cell sorter (BD Biosciences). The threshold for GFP-positive cells was set using dissociated neurons from a non–GFP-expressing WT strain, *Canton S*. The same gating parameters were used to sort other genotypes in the experiment. GFP-positive cells were collected directly in Trizol and then frozen immediately in dry ice until further processing.

##### RNA isolation and qRT-PCR from sorted cells.

Approximately 1200 GFP cells were collected per sample in 500 μl Trizol (Thermo Fisher Scientific) and frozen in dry ice until processing. RNA was isolated following the manufacturer's protocol using glycogen as a carrier (total 5 μg per sample). Isolated RNA was reconstituted in 8 μl nuclease free water, and all of it was used for making cDNA. cDNA synthesis was performed using the SMART-Seq v4 Ultra Low Input RNA Kit (Clontech) following the manufacturer's instructions. Ten cycles of PCR were performed for amplification. Three independent sets of sorted cells were used for each genotype. qPCRs were performed in a total volume of 10 μl with Kapa SYBR Fast qPCR kit (KAPA Biosystems) on an ABI 7500 fast machine (Applied Biosystems). Technical duplicates were performed for each qPCR. A melt analysis was performed at the end of the reaction to ensure the specificity of the product. The fold change of gene expression in any experimental condition relative to control was calculated as follows: 2^−ΔΔCt^, where ΔΔCt = (Ct (target gene) − Ct (housekeeping gene)) _Expt._ − (Ct (target gene) − Ct (housekeeping gene)) _Control_. *Act5c* (see Fig. 7), *rp49* and *tubulin* (Table 2) were used as housekeeping gene controls, and all three yielded similar results. All primer sequences are listed in Table 3.

**Table 2.** Fold changes in mRNA upon *dStim* knockdown in VGN6341 neurons*^a^*

Gene	Normalized to *rp49*					Normalized to *tubulin*				
	*Control*		*dStim KD*		*p* value	*Control*		*dStim KD*		*p* value
	Mean FC	SEM	Mean FC	SEM		Mean FC	SEM	Mean FC	SEM	
*Act5c*	1.01	0.07	1.01	0.05	0.93	1.01	0.08	0.92	0.13	0.58
*itpr*	1.01	0.07	0.79	0.07	0.11	1.00	0.03	0.83	0.19	0.43
*Stim*	1.00	0.07	0.58	0.02	0.00	1.02	0.15	0.54	0.10	0.05
*mAChR*	1.04	0.20	0.41	0.03	0.04	1.02	0.16	0.42	0.09	0.03
*NaCP60E*	1.04	0.23	0.52	0.06	0.09	1.03	0.18	0.55	0.14	0.10
*Hk*	1.08	0.29	0.44	0.02	0.10	1.06	0.26	0.46	0.10	0.09
*eag*	1.05	0.21	0.49	0.04	0.06	1.06	0.26	0.46	0.10	0.09
*cac*	1.00	0.06	0.63	0.15	0.08	1.01	0.13	0.61	0.23	0.20
*ca-alpha1D*	1.00	0.06	0.49	0.04	0.00	1.02	0.14	0.45	0.10	0.03
*VGlut*	1.01	0.12	0.58	0.07	0.03	1.01	0.10	0.58	0.07	0.02

*^a^*Fold changes normalized to two housekeeping genes: *rp49* and *tubulin. p* values obtained from a two-tailed *t* test. FC, Fold change; KD, knockdown. *n* = 3.

**Table 3.** List of primer sequences

Gene	Forward (5′ > 3′)	Reverse (5′ > 3′)
*Act5c*	GTCCACCTTCCAGCAGATG	CCTCCTCCAGCAGAATCAAG
*Ca-alpha1D*	GCGAATGCCATTAACTATGACAAC	ACTCGGAGTCGCAGTATTTACC
*cac*	TGTTCGATTGCGTCGTGAAC	TGGCACTCTGCGGAAGTATG
*eag*	AGGATGTTTCCCTGCTCGTG	TGGGTCACTAACAACCTCGC
*Hk*	CTGTTCGACATCTCGGAGGC	GGTGCTCCAGTAGACCTTCG
*itpr*	CCAGGGTTTGCGAAATGGC	CAGGTCGTCTTCAGAATGGC
*mAChR*	ATGACACCTGGCGACGTCC	CGCAATGCACCACTCCTTG
*NaCP60E*	GACTTTTCCGAGCTACGGGG	TTAAGACAGCCGTCCTCACG
*rp49*	CGGATCGATATGCTAAGCTGT	GCGCTTGTTCGATCCGTA
*slif*	GCTGGACCTGAACACATGGA	TGCGGCTCCTACTTATCTGC
*Stim*	GTACGCTAGATCATGGCCCG	CGTTGTGAGGCAACATTGGG
*VGlut*	CTGTGTTCATTTGGTTGGCTGC	GATCCGTGTTGGTAATGGCAC
β*-tubulin*	CCAAGGGTCATTACACAGAGG	ATCAGCAGGGTTCCCATACC

##### RNA isolation and qRT-PCR for slif.

Body wall preparations from the anterior half of the larvae were obtained by dissecting the skin in PBS prepared in double-distilled water treated with diethyl pyrocarbonate (Sigma-Aldrich) and washing to remove any remnant fat body. Body walls from five larvae were pooled for one sample, and these were homogenized in 500 μl TRIzol (Thermo Fisher Scientific) by vortexing immediately after dissection. At least three biological replicate samples were performed for each genotype. After homogenization, the sample was kept on ice and processed within 30 min or stored at −80°C until processing for up to 4 weeks. RNA was isolated following the manufacturer's protocol. Purity of the isolated RNA was estimated by NanoDrop spectrophotometer (Thermo Fisher Scientific), and integrity was determined by running it on a 1% Tris-EDTA agarose gel.

Approximately 500 ng of total RNA was used per sample for cDNA synthesis. DNase treatment and first strand synthesis were performed as described previously (Richhariya et al., 2017). qPCRs were performed in a total volume of 10 μl with Kapa SYBR Fast qPCR kit (KAPA Biosystems) on an ABI QS3 fast machine (Applied Biosystems). Technical duplicates were performed for each qPCR. A melt analysis was performed at the end of the reaction to ensure the specificity of the product. The fold change of gene expression in any experimental condition relative to the RNAi control was calculated as 2^−ΔΔCt^. *rp49* was used as the housekeeping gene control.

##### Preference assay.

To record larval preference, arginine was obtained from Sigma-Aldrich; 1% agarose solution was prepared and cooled to <50°C before adding the desired quantity of arginine to reach the final concentration of 20 mm. The plate was divided into four agarose quadrants: two with and two without arginine. The quadrants were diametrically opposite to one another. To visually differentiate the quadrants, we used Bromophenol blue on the agarose only quadrants. To ensure that preference was not affected by color, we also performed experiments where the color was mixed in the arginine quadrants, and we did not observe any difference in preference. Plates were prepared fresh ∼2 h before the behavioral assays were performed. Third instar larvae were collected, rinsed with double-distilled water, and starved for at 1.5–2 h on 1% agarose. Groups of 20 animals were placed at the center of each Petri dish under diffuse light. The plates were not covered, and any larvae that came out were ignored for final analyses. We scored for preference at 10 min after addition of the larvae. For optogenetic inhibition experiments, we additionally illuminated the arena with a green LED (Thorlabs) with a central wavelength of 525 nm. Groups of at least 15 animals were placed, and larvae were scored for assessing preference index at 8 min after the green LED being turned on as well as 8 min after the green LED being turned off.

The arena was custom-designed from Styrofoam, and the videos were acquired using a PiCamera coupled to a Raspberry Pi 3.

##### Data representation and statistics.

**Figure 1.**
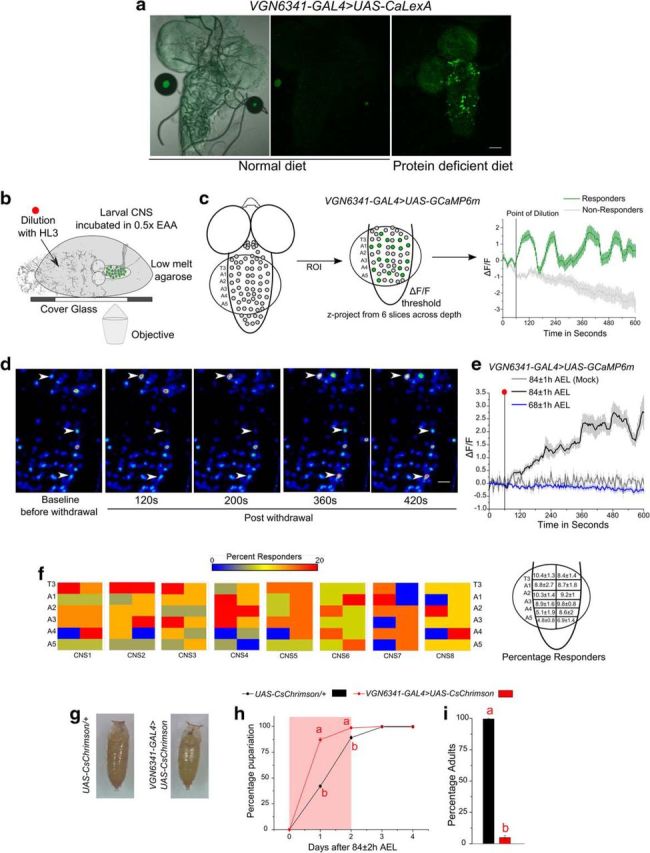
Glutamatergic neurons in larval VG respond to acute amino acid deprivation. ***a***, Confocal images from the whole CNS of third instar larval brains where *UAS-CaLexA* has been driven using *VGN6341-GAL4* on a PDD for 12 h. Scale bar, 50 μm. ***b***, Schematic of the preparation used to observe calcium transients in *VGN6341-GAL4*-marked glutamatergic neurons upon amino acid withdrawal. For details, see Materials and Methods. ***c***, Schematic depicting how cells from T3–A5 are imaged with a sample response of cells classified as responders and nonresponders. ***d***, Images of a sample VG across different times after withdrawal of amino acids. Arrowheads indicate responder cells. ***e***, Line plots of calcium transients observed in glutamatergic neurons marked by *VGN6341-GAL4* when EAAs were withdrawn from CNSs of corresponding age. Red dot indicates the point of withdrawal of amino acids. The transients are from cells that responded and crossed an arbitrary threshold of a minimum change (ΔF/F) of 1.5 after withdrawal as described in Materials and Methods. ***f***, Heatmaps showing distribution of percentage responders in segments T3–A5 from control CNS preps in response to withdrawal of EAA. Right, Diagram represents average responders (±SEM) from each hemisegment. ***g***, Representative pictures of puparia when either glutamatergic neurons marked by *VGN6341-GAL4* or a control genotype were optogenetically activated on ND. ***h***, ***i***, Rate of pupariation and percentage adults, respectively, on ND, when glutamatergic neurons marked by *VGN6341-GAL4* were optogenetically activated for 2 d after 84 ± 2 h AEL. Pupariation at day 1 was significantly different with *p* = 1.14 × 10^−07^ and at day 2, *p* = 5.98 × 10^−05^, using two-tailed Student's *t* test. ***i***, Percentage adults was significantly lower with *p* = 6.68 × 10^−20^, using two-tailed Student's *t* test. Bars with the same alphabet represent statistically indistinguishable groups. Exact *p* values are provided in Figure 1-1.

All bar graphs and line plots represent means, and error bars indicate SEM. In the box plots, horizontal lines in the box indicate medians, crosses indicate the means, box limits indicate the 25th and 75th percentiles, whiskers extend 1.5 times the interquartile range from the 25th and 75th percentiles, individual data points are represented as open circles, and the numbers below indicate the *n* number for each box. All statistical tests are mentioned in the figure legends and were performed using Origin 8.0. Fig. 1-1 has all statistical tests performed for each figure and their exact *p* values.

## Results

### Ca^2+^ transients in glutamatergic neurons encode environmental arginine levels

Glutamatergic neurons located in segments T3-A5 of the VG marked by the *vglut^VGN6341GAL4^* (*VGN6341-GAL4* henceforth) are integral for the decision to pupariate on a PDD (Jayakumar et al., 2016). To test whether *VGN6341-GAL4*-marked glutamatergic interneurons in the CNS of third instar larvae respond to the loss of dietary protein, we expressed in them an activity-dependent reporter, CaLexA (Masuyama et al., 2012). CNSs from larvae placed in a PDD showed a subset of CaLexA-positive neurons, whereas larvae on a ND did not, indicating that loss of dietary protein activates a set of glutamatergic neurons in the VG (Fig. 1*a*).

10.1523/JNEUROSCI.1163-18.2018.f1-1 Figure 1-1 **File contains exact p-values for all statistical tests performed in this paper**. Download Figure 1-1, XLSX file

**Figure 2.**
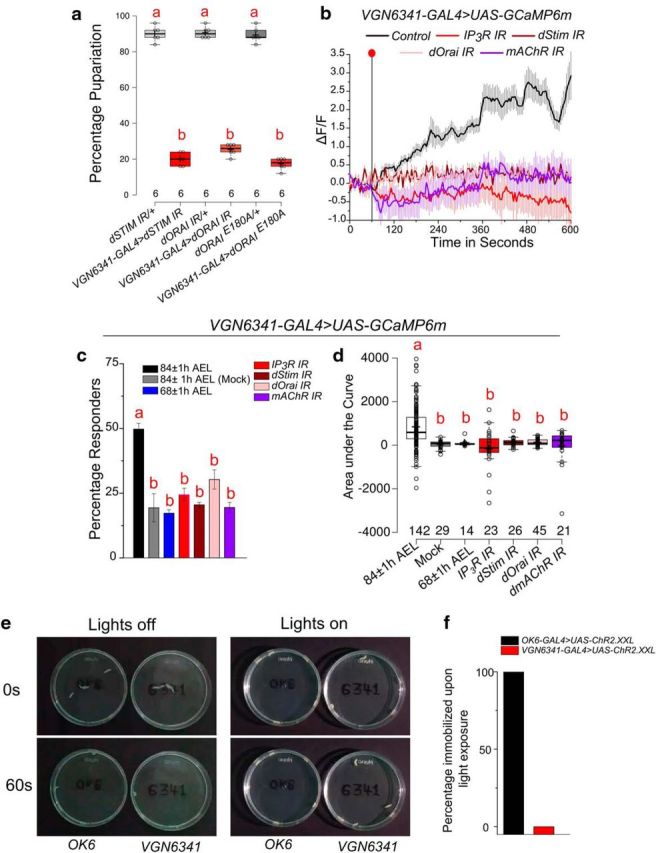
SOCE in larval glutamatergic neurons is important for pupariation under amino acid deprivation. ***a***, Box plots represent percentage pupariation on PDD of the indicated genotypes where intracellular calcium signaling was perturbed in glutamatergic neurons. One-way ANOVA: *F*_(5,30)_ = 652.2215, *p* < 0.05; with *post hoc* Tukey's Multiple Comparison Test (MCT). ***b***, Line plots of calcium transients observed in glutamatergic neurons marked by *VGN6341-GAL4* when EAAs were withdrawn from CNS of indicated genotypes. Red dot indicates the point of withdrawal of EAA. The transients are from cells that responded above an arbitrary threshold of ΔF/F ≥ 1.5 after withdrawal, as described in Materials and Methods. The control genotype trace is from the same experiments as shown in Figure 1*e*. ***c***, ***d***, Percent responders, one-way ANOVA: *F*_(6,31)_ = 16.1339, *p* < 0.05; with *post hoc* Tukey's MCT and (***d***) area under the curve from the line plots shown in Figure 1*e* and 2*b*, one-way ANOVA: *F*_(6,255)_ = 9.25345, *p* < 0.05; with *post hoc* Tukey's MCT. ***e***, Images showing the location of larvae of the indicated genotypes at the indicated time points in either absence or presence of white light. ***f***, Bars represent percentage “freezing behavior” upon exposure to light from the indicated genotypes. Bars and boxes with the same alphabet represent statistically indistinguishable groups. Exact *p* values are provided in Figure 1-1.

To test the real-time effect, if any, of amino acid withdrawal on *VGN6341-GAL4*-marked glutamatergic interneurons calcium transients were measured from semi-intact *ex vivo* preparations. Here, an artificial environment of nutrient withdrawal was created by dilution of amino acids (see Materials and Methods; Fig. 1*b*,*c*). Withdrawal of EAAs from the environment of third instar larval CNS yielded robust calcium transients in ∼50% of marked neurons as reported by the calcium sensor GCaMP6m (Figs. 1*d*,e, 2*c*). To understand whether there existed a specific subset of glutamatergic interneurons in segments T3-A5 that respond consistently to amino acid withdrawal, we quantified the responsive cells in each hemisegment. Interestingly, cells that responded within the T3-A5 region did not explicitly follow an anatomical bias for any segment or hemisegment across CNSs from multiple larvae (Fig. 1*f*).

The observed calcium transients were specific to mid-third instar larvae, as they were not elicited from glutamatergic neurons in the VG upon EAA withdrawal from the CNS of second instar larvae (Figs. 1*e*, 2*c*,*d*). These data indicate that the responses for sensing nutrient withdrawal are stage-specific and are possibly required for the decision to pupariate. In agreement with this idea, optogenetic activation of *VGN6341-GAL4*-marked neurons of mid-third instar larvae, on an ND, resulted in premature pupariation (Fig. 1*g–i*), where the puparia appeared visually similar to controls (Fig. 1*g*). However, they were not viable (Fig. 1*i*), possibly because they lacked other physiological parameters for normal pupariation.

We hypothesized that the observed calcium transients in glutamatergic neurons encode a neural decision to continue development and pupariate under nutrient stress. To understand the molecular basis of the observed neuronal calcium transients, we first tested the nature of calcium signaling required for generating these transients. We previously identified intracellular calcium signaling in this subset of glutamatergic neurons as essential for pupariation on a PDD (Jayakumar et al., 2016). Indeed, calcium transients observed upon EAA withdrawal in glutamatergic neurons in the VG were significantly attenuated upon knockdown of components of intracellular calcium signaling and SOCE, such as the IP_3_R, dSTIM, and dOrai (Fig. 2*a*,*c*,*d*). The loss of transients also correlated with loss of pupariation on PDD in these genotypes (Fig. 2*b*).

Glutamatergic neurons in the *Drosophila* VG include both motor neurons and interneurons. To test whether *VGN6341-GAL4*-marked neurons were indeed interneurons and did not include many motor neurons, an optogenetic activation experiment was performed. Disruption of motor neuron activity followed by freezing behavior has been demonstrated earlier upon optogenetic activation of motor neurons (Dawydow et al., 2014). Similar freezing behavior was evident upon optogenetic activation of larval motor neurons marked by *OK6-GAL4* (Fig. 2*e*,*f*). However, normal larval movement was evident upon optogenetic activation of the *VGN6341-GAL4* neurons (Fig. 2*e*,*f*), indicating that a majority are likely to be interneurons and are not involved in larval locomotion.

**Figure 3.**
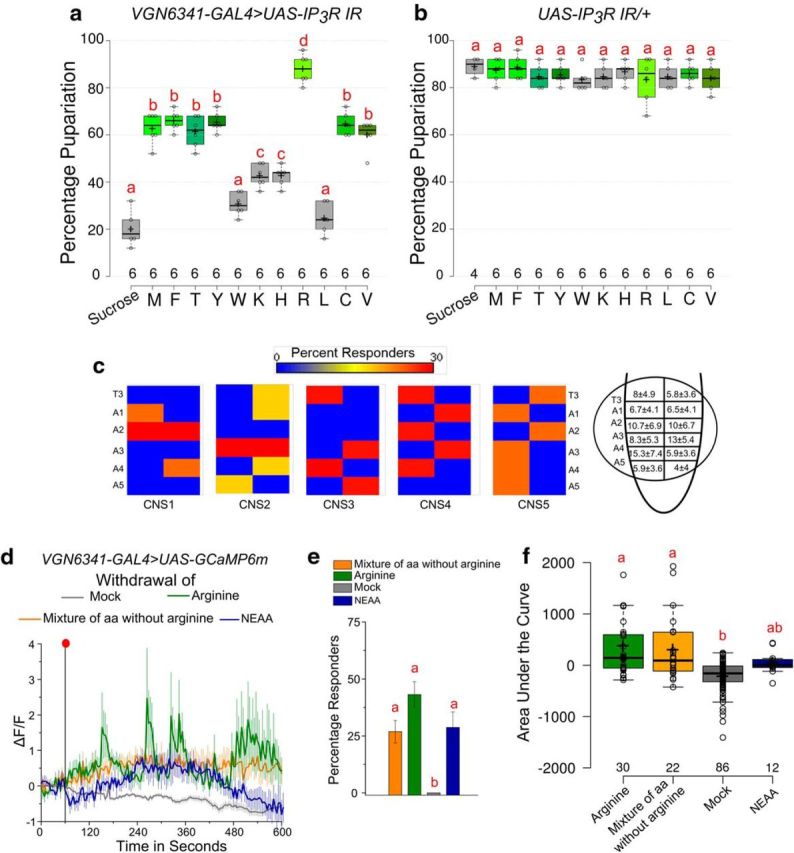
Arginine is critical amino acid for pupariation. ***a***, ***b***, Percentage pupariation of the indicated genotypes on PDD upon supplementation with indicated specific amino acids. Numbers indicate batches of 25 larvae each. One-way ANOVA: *F*_(11,60)_ = 78.45015, *p* < 0.05; with *post hoc* Tukey's MCT for ***a*** and one-way ANOVA: *F*_(11,58)_ = 0.73875, *p* = 0.697; with *post hoc* Tukey's MCT for ***b***. ***c***, Heat maps showing the distribution of percentage of cells that responded to withdrawal of arginine in segments T3–A5 from five control CNS preparations. Right, Diagram represents the mean and SEM of number of responding cells in each indicated hemisegment as calculated from the five CNS preparations. ***d***, Line plots of calcium transients in responding CNS cells observed upon withdrawal of arginine, a mixture of EAAs lacking arginine, a mixture of non-EAAs or no withdrawal (mock-withdrawal). The mock-withdrawal trace is from all cells as none of them crossed the threshold. ***e***, ***f***, Percent responders and area under the curve quantified from the line plots shown in ***d***. One-way ANOVA: *F*_(3,14)_ = 16.0049, *p* < 0.05; with *post hoc* Tukey's MCT for ***e*** and one-way ANOVA: *F*_(3,146)_ = 14.00764, *p* < 0.05; with *post hoc* Tukey's MCT for ***f***. Bars and boxes with the same alphabet represent statistically indistinguishable groups. Exact *p* values are provided in Figure 1-1.

Next, we tested whether specific amino acids were required in the diet for pupariation of larvae with IP_3_R knockdown in neurons of the VGs. We tested supplementation of PDD with all EAAs (except glutamate). Strikingly, supplementation of arginine alone was sufficient for a complete rescue of the pupariation deficit observed upon reduced intracellular calcium signaling in larvae at 84 h AEL (Fig. 3*a*,*b*). Withdrawal of arginine from the CNS of 84 h AEL larvae resulted in robust calcium transients in ∼42% cells compared with 0% in cells with no change in environmental arginine levels (Fig. 3*d–f*). Moreover, withdrawal of a mixture of EAAs, except arginine, elicited a less pronounced response from just 27% cells and withdrawal of a mixture of non-EAAs elicited a further attenuated response (Fig. 3*d–f*). Cells that responded to arginine withdrawal did not exhibit an obvious anatomical focus within T3-A5 segments of the VG (Fig. 3*c*) similar to what was observed upon EAA withdrawal. These data indicate that, although withdrawal of all amino acids together yields a strong response, withdrawal of arginine alone is sufficient to elicit calcium transients in glutamatergic interneurons.

**Figure 4.**
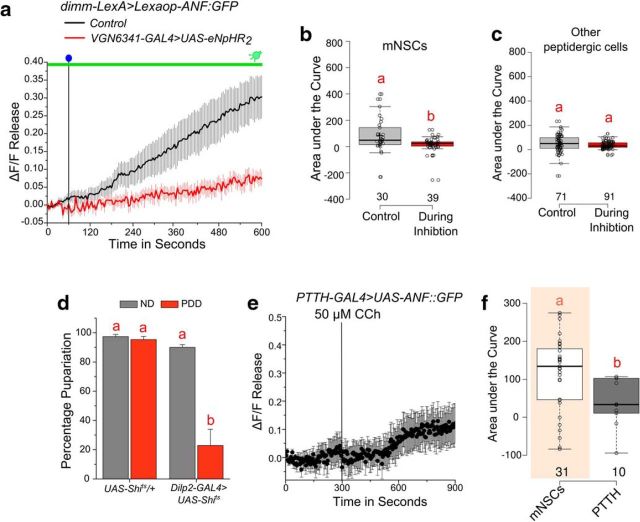
Withdrawal of amino acid in the media can induce glutamatergic neuron-dependent peptide release. ***a***, Line plots of peptide release from mNSCs marked by *dimm-LexA*. Peptide release was measured by decrease in fluorescence of the atrial natriuretic factor linked to GFP (ANF::GFP) following withdrawal of arginine from CNS with and without optical inhibition of glutamatergic neurons with eNpHR2. Blue dot indicates the point of withdrawal of arginine. Green line indicates the duration of inhibition by eNpHR2. ***b***, ***c***, Box plots represent area under the curves quantified from the peptide release response of mNSCs (***b***) and non-mNSC peptidergic cells (***c***). The release was found to be significantly lower during inhibition from the mNSC in ***b*** (*p* = 0.00525, two-tailed Student's *t* test), whereas the release from the non-mNSC (***c***) cells was not (*p* = 0.13651, two-tailed Student's *t* test). All data are from a minimum of four CNSs of the individual genotypes. ***d***, Percentage pupariation of the indicated genotypes on indicated diets concurrent with a temperature shift from permissive (18°C) to restrictive temperature (29°C), thereby blocking peptide release on PDD. Two-way ANOVA: *F*_(3,21)_ = 41.09651, *p* < 0.05; with *post hoc* Tukey's MCT. ***e***, Line plot showing peptide release from *PTTH-GAL4*-marked cells assayed by expression of the ANF::GFP construct and measured upon stimulation with 50 μm carbachol. ***f***, Box plots represent release as estimated by area under the curves for either mNSCs or from PTTH cells (*p* = 0.02858, two-tailed Student's *t* test). The mNSC data are the same as in Jayakumar et al. (2016; their Fig. 6*a*). Bars and boxes with the same alphabet represent statistically indistinguishable groups. Exact *p* values are provided in Figure 1-1.

Glutamatergic interneurons regulate peptide release from medial neurosecretory cells (mNSC) required for pupariation on PDD (Jayakumar et al., 2016). We thus tested the physiological relevance of transients observed upon loss of arginine. Peptide release was monitored with ANF::GFP from all cells marked by *dimm-LexA* that specifically drives expression in neuropeptidergic cells (Rao et al., 2001; Jayakumar et al., 2016). Withdrawal of arginine resulted in peptide release from numerous peptidergic cells in the larval CNS (Fig. 4*a*). Peptide release from all the mNSCs elicited by withdrawal of arginine was attenuated by optogenetic inhibition of activity in glutamatergic neurons marked by *VGN6341-GAL4* (Fig. 4*a*,*b*). Similar inhibition of peptide release was not observed from other peptidergic cells in the CNS (Fig. 4*c*). Thus, although amino acid withdrawal elicits peptide release from various peptidergic cells, dependence of peptide release on glutamatergic neuron activity appears limited to the mNSCs (Fig. 4*b*). Earlier, we observed that release of *Dilp2* from the mNSCs is diet-dependent and stimulated by activation of *VGN6341-GAL4* neurons (Jayakumar et al., 2016). In agreement with this, loss of pupariation was observed in larvae on PDD upon blocking peptide release from *Dilp2*-producing cells of the mNSCs. On an ND, however, pupariation does not require peptide release from the mNSCs (Fig. 4*d*). These results demonstrate that loss of dietary arginine stimulates calcium transients in glutamatergic interneurons and these transients stimulate peptide release from the mNSCs.

The mNSCs are known to send projections to the ring gland (Cao and Brown, 2001), and possibly stimulate synthesis of the molting hormone ecdysone (Jayakumar et al., 2016). However, on a ND, it is well established that the prothoracicotropic hormone (PTTH), produced by a pair of bilateral neurons in the central brain, has an essential role in ecdysone production (McBrayer et al., 2007). Previous work has demonstrated that stimulation of glutamatergic interneurons marked by *VGN6341-GAL4* elicits peptide release from mNSCs, as monitored by ANF::GFP (Jayakumar et al., 2016). However, in a similar experiment, peptide release from the PTTH neurons was significantly lower than from the mNSCs (Fig. 4*e*,*f*). Together, these data suggest that peptide release from PTTH neurons is not stimulated directly by *VGN6341-GAL4*-marked glutamatergic interneurons. However, they do not rule out either an independent role of PTTH neurons or indirect stimulation of PTTH neurons by mNSC secreted peptides, in pupariation on PDD.

### Peptidergic modulation of Ca^2+^ transients upon amino acid withdrawal

**Figure 5.**
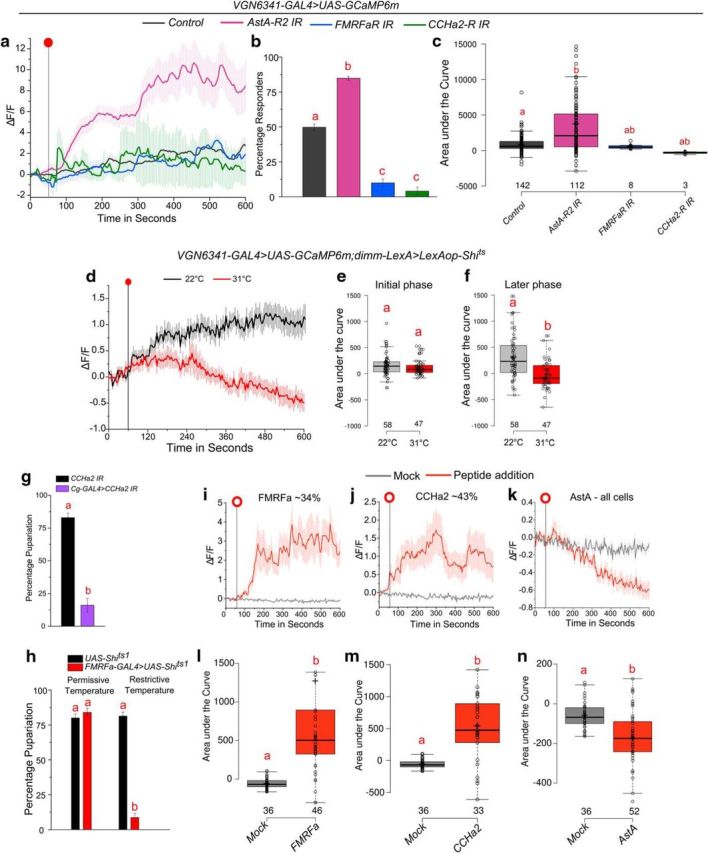
Neuropeptides modulate calcium transients and pupariation during protein deprivation. ***a***, Line plots of calcium transients from larval CNSs observed in *VGN6341-GAL4*-marked glutamatergic neurons upon withdrawal of EAA in animals with the indicated neuropeptide receptor knockdowns. Red dot indicates the point of withdrawal of EAA. The transients are from cells that responded above an arbitrary threshold of ΔF/F ≥ 1.5 after withdrawal, as described in Materials and Methods. The control genotype trace is from the same experiments as shown in Figure 2*b*. ***b***, Bars represent percentage of cells that responded to EAA withdrawal above the threshold from the indicated genotypes as shown in ***a***. One-way ANOVA: *F*_(3,19)_ = 212.26, *p* < 0.05; with *post hoc* Tukey's MCT. ***c***, Box plots represent area under the curve from the graph in ***a***. One-way ANOVA: *F*_(3,225)_ = 9.32574, *p* < 0.05; with *post hoc* Tukey's MCT. ***d***, Line plots of calcium transients from larval CNS observed in *VGN6341-GAL4*-marked glutamatergic neurons upon withdrawal of EAA in animals upon blocking peptide release either at restrictive (31°C) or permissive (22°C) temperatures. Red dot indicates the point of withdrawal of EAA. The transients are from cells that responded above an arbitrary threshold of ΔF/F ≥ 1.5 after withdrawal, as described in Materials and Methods. ***e***, ***f***, Box plots represent area under the curve from the graph in ***d***, for the initial phase (60–300 s) were not significant (*p* = 0.15686, two-tailed Student's *t* test) but were significant in the later phase (300–600 s) (*p* = 4.30 × 10^−06^, two-tailed Student's *t* test). ***g***, Bars represent percentage pupariation observed when CCHa2 knockdown was performed in the fat body (*p* = 3.69 × 10^−05^, two-tailed Student's *t* test). ***h***, Bars represent percentage pupariation observed when FMRFa release is either permitted (*p* = 0.35576, two-tailed Student's *t* test) or inhibited from the CNS under conditions of nutrient deprivation (*p* = 3.75 × 10^−08^, two-tailed Student's *t* test). ***i–k***, Line plots of calcium transients observed upon addition of 5 μm of the corresponding neuropeptide. Open red circles represent the point of addition of neuropeptide. Percent cells that responded are indicated on top of the graphs. For AstA, all cells were below the threshold. The mock trace was obtained from all cells, and the same trace is shown in the three graphs. All data are from a minimum of five CNSs of the individual genotypes. ***l–n***, Box plots represent area under the curve from graphs in ***i***, ***j***, and ***k***, respectively, with *p* = 3.41228 × 10^−05^ (***l***), *p* = 0.000527221 (***m***), and *p* = 0.000535367 (***n***) (two-tailed Student's *t* test). Boxes and bars with the same alphabet represent statistically indistinguishable groups. Exact *p* values are provided in Figure 1-1.

Reduction in environmental arginine levels elicited peptide release from various neuropeptidergic cells in the brain (Fig. 4*a*,*b*). A screen for receptors on glutamatergic neurons in the VG, which modulate pupariation on PDD, identified several neuropeptide receptors as well as the mAChR, all of which couple with intracellular Ca^2+^ signaling (Jayakumar et al., 2016). Calcium transients upon EAA withdrawal were lost upon knockdown of mAChR (Fig. 2*a*,*c*,*d*). To test whether neuropeptide modulation upon amino acid withdrawal is additionally responsible for the observed calcium transients, we knocked down expression of three neuropeptide receptors (i.e., FMRFaR, CCHa2-R, and AstA-R2) identified in the screen for pupariation deficits on PDD (Jayakumar et al., 2016). The number of cells that responded to withdrawal of amino acids fell to <10% when either FMRFaR or CCHa2-R were knocked down in glutamatergic neurons (Fig. 5*a–c*). Response amplitudes of the responding cells were, however, comparable with control cells (Fig. 5*a*). On the other hand, knockdown of AstA-R2 resulted in both an increase in the percentage of responding cells and a significant increase in response amplitude (Fig. 5*a*,*b*). Thus, EAA withdrawal-mediated calcium transients in glutamatergic neurons are modulated by multiple neuropeptide receptors in addition to cholinergic inputs through the mAChR.

The ability of neuropeptides to stimulate calcium responses in the glutamatergic neurons of the VG was tested next. Calcium transients in glutamatergic interneurons were significantly attenuated upon EAA withdrawal when peptide release was blocked from neuropeptide-secreting neurons (Fig. 5*d–f*). Interestingly, blocking of peptide release did not affect the response of glutamatergic interneurons immediate to the event of amino acid deprivation, but there was a significant effect on the maintenance of calcium transients (Fig. 5*e*,*f*). Direct addition of either FMRFa or CCHa2 yielded calcium transients from 34% and 43% of *VGN6341-GAL4*-marked glutamatergic neurons, respectively, in the VG (Fig. 5*i*,*j*,*l*,*m*). Consistent with AstAR knockdown data (Fig. 5*a*), addition of AstA peptide led to a significant decay in the calcium response (Fig. 5*k*,*n*). Glutamatergic neurons in the VGs thus receive neuropeptide signals through FMRFa, AstA, CCHa2, and possibly other neuropeptides in response to amino acid withdrawal.

The role of CCHa2 and FMRFa for pupariation on PDD was tested next. Knockdown of CCHa2 specifically in the fat body led to pupariation deficits (Fig. 5*g*). Similarly, blocking vesicle recycling, and therefore peptide release from established Tv FMRFa neurons in the larval CNS (Santos et al., 2007), resulted in pupariation deficits upon protein deprivation (Fig. 5*h*). These data support the idea that glutamatergic neurons in the VG integrate information regarding protein deprivation by neurohormonal peptide release from multiple internal tissues in addition to the external environment.

### *ppk* neurons express *slimfast* for sensing arginine as a proxy for diet quality

**Figure 6.**
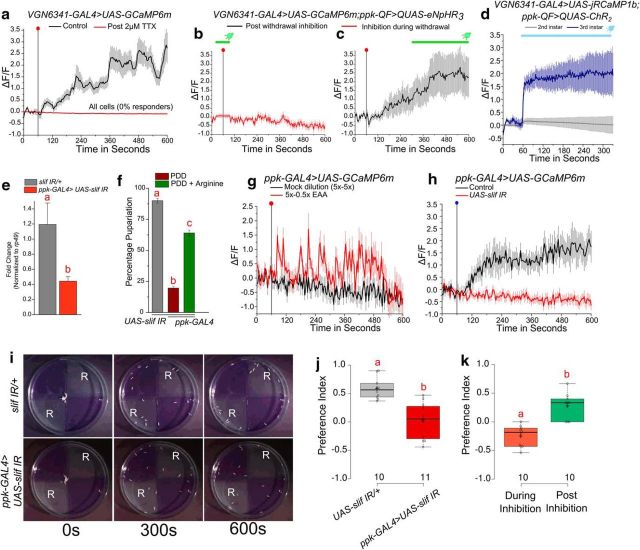
*slif* in ppk neurons senses arginine as a proxy for nutritional quality. ***a***, Line plots represent calcium transients observed from *VGN6341-GAL4*-marked glutamatergic neurons upon withdrawal of EAA from CNS with and without application of 2 μm TTX. Responses from all cells upon TTX application have been plotted. The control data are the same as used in Figure 2*b*. ***b***, ***c***, Line plots of calcium transients from glutamatergic neurons observed upon withdrawal of EAA when inputs from *ppk-GAL4*-marked sensory neurons were inhibited either concurrent with withdrawal (***b***) or after withdrawal (***c***). Red dot indicates the point of withdrawal of EAA. Green line on top of the graph indicates duration of inhibition. The transients are from all cells in ***b*** and from the responding cells (∼55%) in ***c***. Responding cells were classified by an arbitrary threshold of ΔF/F ≥1.5 after withdrawal, as described in Materials and Methods. All data are from a minimum of five CNSs of the individual genotypes and each at least 57 cells. ***d***, Line plots of calcium transients from glutamatergic neurons observed upon activating *ppk* neurons in either second (68 ± 2 h AEL) or third instar larvae (84 ± 2 h AEL). Blue line on top of the graph indicates duration of activation. ***e***, Bars represent fold change in mRNA levels of *slif* normalized to *rp49* in the indicated genotypes (*p* = 0.021, two-tailed Student's *t* test). ***f***, Bars represent percentage pupariation in the indicated genotypes, one-way ANOVA: *F*_(2,19)_ = 390.6136, *p* < 0.05; with *post hoc* Tukey's MCT. ***g***, Line plots represent calcium transients observed in *ppk* neurons upon withdrawal of EAA from semi-intact preparations. Red dot indicates the point of withdrawal of EAA. The transients shown are from all cells. ***h***, Line plots represent calcium transients observed upon withdrawal of arginine from semi-intact preparations in control as well as knockdown of *slif* using *ppk-GAL4.* Blue dot indicates the point of withdrawal of arginine. The transients shown are from all cells. ***i***, Representative images from the preference assay at the indicated times in control animals (*slif IR*/+) and animals with *slif* knockdown in *ppk-GAL4*-expressing sensory neurons. ***j***, ***k***, Box plots of the preference index calculated either at the end of 10 min in animals of the indicated genotypes (*p* = 0.000105775, two-tailed Student's *t* test; ***j***) or during and after real-time optical inhibition of *ppk-GAL4* neurons expressing eNpHR3 (*p* = 1.6009 × 10^−05^, two-tailed Student's *t* test; ***k***), where the numbers indicate the number of batches of 20 larvae that were tested. Bars and boxes with the same alphabet represent statistically indistinguishable groups. Exact *p* values are provided in Figure 1-1.

Next, we tested whether glutamatergic neurons in the VG sense loss of arginine directly. For this, we bath-applied TTX to inhibit polysynaptic inputs. Upon withdrawal of EAA in the presence of 2 μm TTX, calcium transients in the glutamatergic neurons were abolished (Fig. 6*a*), indicating that these glutamatergic neurons respond to loss of arginine indirectly. A potential class of input neurons, implicated from our earlier study (Jayakumar et al., 2016), were the Class IV multidendritic neurons labeled by *ppk-GAL4*, which are cholinergic (Imlach et al., 2012). Optogenetic inhibition of these multidendritic neurons with a light-activated chloride pump (halorhodopsin; eNpHR3), concurrent with withdrawal of amino acids, abolished the transients (Fig. 6*b*), whereas inhibition after the onset of transients failed to affect the maintenance of transients (Fig. 6*c*). In agreement with previous data (Jayakumar et al., 2016), optogenetic activation of ppk neurons activated glutamatergic neurons marked by *VGN6341-GAL4* in third instar larval CNS. Interestingly, optogenetic activation of *ppk* neuron activation of glutamatergic neurons marked by *VGN6341* was absent in second instar larvae (Fig. 6*d*). These data indicate that cholinergic inputs from Class IV multidendritic neurons initiate the calcium transients upon loss of arginine in third instar larvae. The multidendritic neurons marked by *ppk-GAL4* are nociceptive (Hwang et al., 2007; Zhong et al., 2010), and their ability to sense arginine has not been reported previously.

A possible candidate as a sensor for arginine is the amino acid transporter Slimfast (encoded by the gene *slif*) with a known preference for arginine (Boudko et al., 2015; Croset et al., 2016), but its expression has been reported only in the larval fat body (Colombani et al., 2003). To test for *slif* expression in Class IV multidendritic neurons, *slif* levels were measured from the body wall of control larvae and larvae expressing *slif* RNAi driven by *ppk-GAL4. slif* expression in the body wall was significantly reduced upon knockdown by *ppk-GAL4* in Class IV multidendritic neurons (Fig. 6*e*). Larvae with *slif* knockdown in *ppk* neurons also exhibit significant pupariation deficits on PDD (Fig. 6*f*). The pupariation deficit could be rescued to a significant extent by supplementing PDD with just arginine (Fig. 6*f*). To test whether *ppk* neurons sense arginine directly, we performed withdrawal of either EAA or arginine alone and observed calcium transients under both conditions from *ppk*-labeled neurons in semi-intact preparations (Fig. 6*g*). Importantly, these transients were abrogated by *slif* knockdown in *ppk* neurons (Fig. 6*h*). Together, these data confirm that *slif* is required in Class IV multidendritic neurons for the physiological response to loss of arginine in the diet.

*Drosophila* larvae exhibit an innate preference for arginine (Croset et al., 2016). Interestingly, knockdown of *slif* by *ppk-GAL4* attenuated this innate preference (Fig. 6*i*,*j*). Requirement of Class IV multidendritic neurons in sensing and mediating the response to arginine in real-time was confirmed by inhibiting *ppk* neurons with halorhodopsin. When activity in *ppk* neurons was inhibited, larvae lost their preference for arginine; whereas upon removal of the inhibition, their innate preference for a diet with arginine was restored (Fig. 6*k*). Thus, *slif* on ppk neurons appears to sense arginine in the environment, in addition to perhaps arginine levels in the hemolymph. Together, these results suggest that arginine functions as a proxy for assessing diet quality in *Drosophila* larvae. This information encodes a real-time behavioral change, as well as a physiological response that controls the developmental decision to pupariate in the absence of arginine.

### IP_3_R-mediated Ca^2+^ release and SOCE regulate neuronal function through gene expression

**Figure 7.**
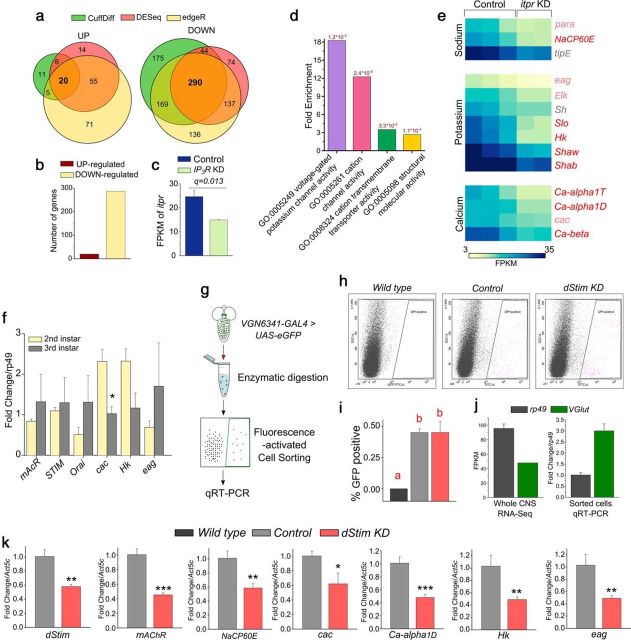
Intracellular calcium signaling through IP3R/SOCE in glutamatergic neurons regulates expression of genes encoding ion channels. ***a***, Venn diagrams representing the number of genes identified as differentially expressed by three independent, indicated methods. *IP_3_R* knockdown majorly leads to downregulation of a set of genes. For list of genes, refer to Figure 7-1. ***b***, Bars represent the number of genes upregulated or downregulated upon knockdown of the *IP_3_R* in the CNS as measured by RNA-seq. ***c***, Bars represent the expression levels of *IP_3_R* in the indicated conditions. *q* value refers to the corrected *p* value obtained from CuffDiff. ***d***, Bars represent the fold enrichment in the number of genes of the indicated GO molecular function categories in the set of genes downregulated upon *IP_3_R* knockdown, compared with all genes in *Drosophila*. Numbers on top of the bars indicate FDR corrected *p* values. This analysis was performed using the Panther GO Slim Molecular Function option. ***e***, Heatmap indicates the fragments per kilobase per million (FPKM) values as a proxy for expression level of the indicated cation channel genes in control (UAS-*IP_3_R IR/+; UAS-dicer2*/+) and *IP_3_R* knockdown (*elav^C155-^GAL4*>*UAS-IP_3_R IR; UAS-dicer2*) conditions. Red labels indicate genes whose expression is significantly altered by *IP_3_R* knockdown identified by all three methods (CuffDiff, DESeq, and edgeR). Pink labels indicate differential gene expression significant by any two methods. Gray labels indicate differential expression of genes that are not significant. ***f***, Bars represent the fold change in expression levels of the indicated genes normalized to *rp49* measured by qRT-PCR from CNSs of second instar larvae to that of third instar (*p* = 0.019984 for *cac*, two-tailed Student's *t* test). ***g***, Diagram representation of the procedure used to sort glutamatergic neurons of interest. ***h***, Representative dot plots of flow cytometric analysis of cell suspensions made from the indicated genotypes. *x* axis indicates the extent of fluorescence; *y* axis indicates a measure of granularity based on the side-scatter. Threshold was set using the nonfluorescent WT and pink dots were collected as GFP-positive cells. ***i***, Bars represent the percentage of GFP-positive glutamatergic cells obtained by FACS from the indicated genotypes. One-way ANOVA: *F*_(2,9)_ = 24.3, *p* < 0.05; with *post hoc* Tukey's MCT. Bars with the same alphabet represent statistically indistinguishable groups. ***j***, Comparison of the levels of *VGlut* in whole CNS versus sorted glutamatergic neurons compared with the housekeeping gene, *rp49. VGlut* expression is enriched in the sorted glutamatergic neurons. ***k***, Bars represent the fold change in expression levels of the indicated genes normalized to *Act5c* measured by qRT-PCR from sorted glutamatergic cells of the control (*VGN6341-GAL4*>*UAS-eGFP*) and *dStim* KD (*VGN6341-GAL4*>*UAS-eGFP; UAS-dStim IR; dcr2*) genotypes; *p* = 0.012 (*dStim*), *p* = 0.003 (mAChR), *p* = 0.023 (*NaCP60E*), *p* = 0.040 (*Hk*), *p* = 0.040 (*eag*), *p* = 0.077 (*cac*), *p* = 0.008 (Ca-α1D). RNA was isolated from ∼1200 sorted neurons and amplified using the SMART-seq method before performing qRT-PCR. *p < 0.1 (two-tailed *t* test). **p < 0.05 (two-tailed *t* test). ***p < 0.01 (two-tailed *t* test). Exact *p* values are provided in Figure 1-1.

Next, we addressed why loss of mAChR and neuropeptide receptor stimulated intracellular calcium signaling through the IP_3_R and dSTIM abrogated the polysynaptic Ca^2+^ transients in VG glutamatergic neurons observed upon withdrawal of EAAs (Fig. 2*b*). IP_3_-mediated Ca^2+^ release is followed by dSTIM/dOrai-mediated SOCE in *Drosophila* neurons (Venkiteswaran and Hasan, 2009; Chakraborty et al., 2016). Both IP_3_-mediated Ca^2+^ release and SOCE alter cytosolic Ca^2+^ levels and affect function in mammalian neurons (Futatsugi et al., 1999; Fujii et al., 2000; Hartmann and Konnerth, 2005), although the precise cellular mechanisms remain to be elucidated. STIM/Orai-mediated SOCE also regulates gene expression in nonexcitable and excitable cells (Feske, 2007; Richhariya et al., 2017). As the calcium transients are stage-specific (Fig. 1*e*), we hypothesized that gene expression changes may underlie the ability of glutamatergic neurons to respond to nutrient stress and loss of Ca^2+^ transients in genotypes affecting intracellular Ca^2+^ signaling (Fig. 2*b*) may arise in part from altered gene expression. To test this, we profiled the transcriptome of the third instar larval CNS with and without knockdown of the *IP_3_R* by RNA-seq. Analysis by three independent methods (see Materials and Methods) identified expression of 20 genes as upregulated and 287 genes as downregulated in *IP_3_R* knockdown larval CNS (Fig. 7*a*,*b*; Fig. 7-1), indicating positive regulation of gene expression by IP_3_-mediated Ca^2+^ signaling. *IP_3_R* knockdown was confirmed by RNA-seq (Fig. 7*c*). Importantly, gene ontology analysis of the 287 downregulated genes revealed significant enrichment in categories of ion transport, membrane depolarization, and synaptic signaling (Fig. 7*d*; Table 4). Indeed, the expression of genes encoding various ion channels was downregulated upon IP_3_R knockdown (Fig. 7*i*). Together, these data suggest that attenuation of calcium signaling through the IP_3_R in the larval CNS of third instar larvae alters neuronal function by change in the expression of ion channel genes. To test whether such expression of ion channels is the reason why glutamatergic interneurons in second instar larval CNS remain unresponsive to EAA withdrawal, we compared expression of selected calcium signaling and ion channel genes between second and third instar larval brains (Fig. 7*f*). The genes tested do not appear to be differentially expressed between the CNS of second and third instar larvae, indicating that the absence of response to EAA withdrawal in the VGs of second instar larvae might not be a consequence of IP_3_R/SOCE-regulated gene expression.

10.1523/JNEUROSCI.1163-18.2018.f7-1 Figure 7-1 **File contains lists of genes that were identified as differentially regulated by all three methods used**. Download Figure 7-1, XLSX file

**Table 4.** GO classification of down-regulated genes*^a^*

GO term	Description	*p* value	FDR *q* value	Enrichment
GO:0008010	Structural constituent of chitin-based larval cuticle	8.27E-18	2.02E-14	15.26
GO:0042302	Structural constituent of cuticle	2.15E-17	2.62E-14	13.37
GO:0005214	Structural constituent of chitin-based cuticle	1.19E-16	9.7E-14	13.53
GO:0005261	Cation channel activity*^b^*	3.34E-10	2.03E-7	7.25
GO:0022843	Voltage-gated cation channel activity*^b^*	7.15E-10	3.48E-7	13.78
GO:0005216	Ion channel activity*^b^*	9.33E-10	3.79E-7	5.89
GO:0022838	Substrate-specific channel activity	1.8E-9	6.26E-7	5.67
GO:0005198	Structural molecule activity	3.1E-9	9.46E-7	3.23
GO:0022836	Gated channel activity	3.64E-9	9.84E-7	7.35
GO:0046873	Metal ion transmembrane transporter activity	7.05E-9	1.72E-6	5.22
GO:0022803	Passive transmembrane transporter activity	1.08E-8	2.39E-6	5.09
GO:0015267	Channel activity*^b^*	1.08E-8	2.19E-6	5.09
GO:0022832	Voltage-gated channel activity*^b^*	1.34E-8	2.51E-6	10.67
GO:0005244	Voltage-gated ion channel activity*^b^*	1.34E-8	2.33E-6	10.67
GO:0008324	Cation transmembrane transporter activity	2.06E-7	3.35E-5	3.55
GO:0015075	Ion transmembrane transporter activity	2.72E-7	4.15E-5	3.00
GO:0022891	Substrate-specific transmembrane transporter activity	6.25E-7	8.95E-5	2.81
GO:0022890	Inorganic cation transmembrane transporter activity	9.3E-7	1.26E-4	3.65
GO:0022892	Substrate-specific transporter activity	2.26E-6	2.9E-4	2.52
GO:0015085	Calcium ion transmembrane transporter activity	7.21E-6	8.78E-4	7.56
GO:0022857	Transmembrane transporter activity	7.67E-6	8.9E-4	2.42

*^a^*GO classification of the molecular function of the genes downregulated upon *IP_3_R* knockdown was performed using Gene Ontology enRIchment anaLysis and visuaLizAtion tool (GORILLA). Enriched categories with associated fold enrichment, *p* value, and FDR corrected *q* value are indicated.

*^b^*Various categories related to voltage-gated ion channels were identified.

**Figure 8.**
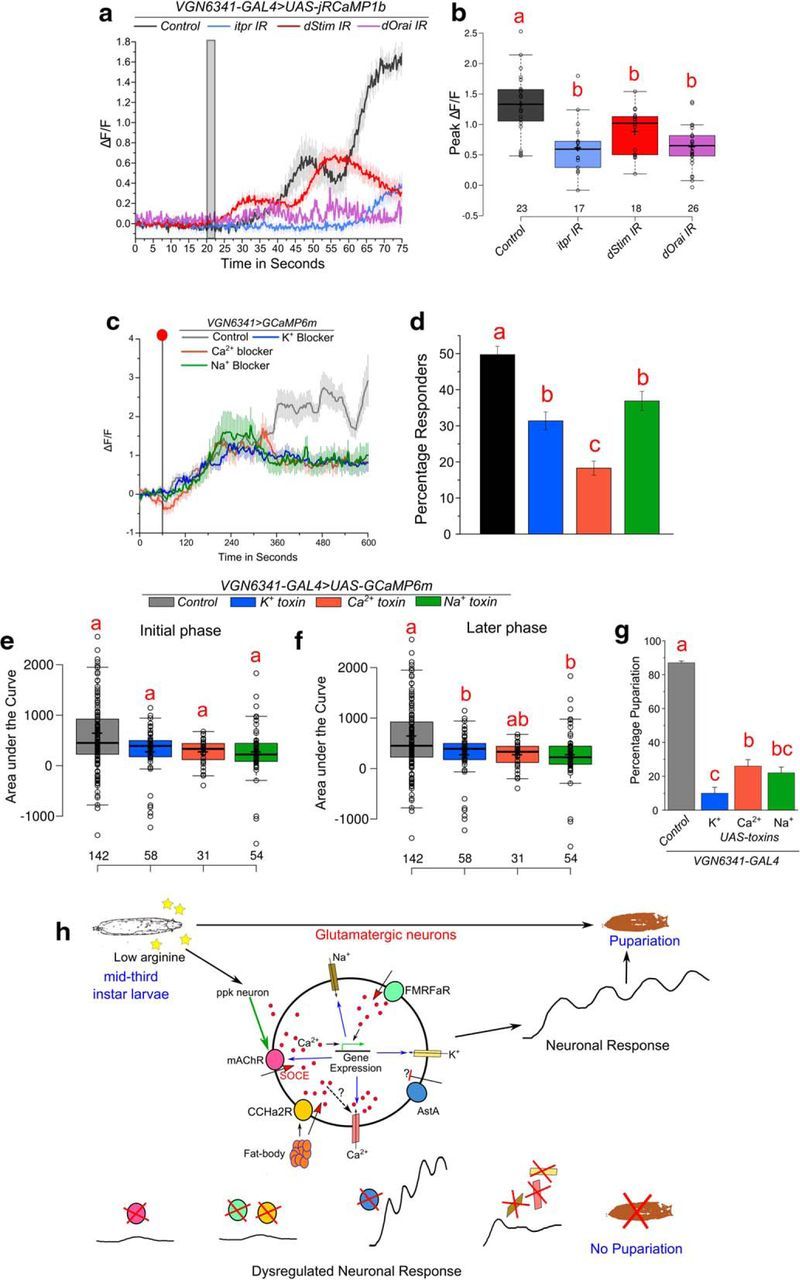
IP_3_R/SOCE in glutamatergic neurons regulates neuronal excitability. ***a***, Line plots represent calcium transients observed upon depolarization by KCl in *VGN6341-GAL4*-marked glutamatergic neurons from CNS of the indicated genotypes. Gray box represents the window of addition of KCl. Responses from all cells have been plotted. ***b***, Box plots indicate the peak change in fluorescence from traces in ***a***. One-way ANOVA: *F*_(3,80)_ = 13.44943, *p* < 0.05; with *post hoc* Tukey's MCT. ***c***, Line plots indicate calcium transients observed upon withdrawal of EAAs in control CNS and in CNS with expression of the indicated toxins in *VGN6341-GAL4*-marked glutamatergic neurons. Red dot indicates the point of withdrawal of EAA. The transients are from cells that responded above an arbitrary threshold of ΔF/F ≥ 1.5 after withdrawal, as described in Materials and Methods. Data are from a minimum of five CNSs of the individual genotypes. Control trace is the same as in Figure 2*b*. ***d***, Percent responders from the trace in ***c***, one-way ANOVA: *F*_(3,23)_ = 30.58001, *p* < 0.05; with *post hoc* Tukey's MCT. ***e***, ***f***, Area under the curve quantified from the trace in Figure 8*c* for the initial phase from 60 to 300 s, one-way ANOVA: *F*_(3,243)_ = 0.28392, *p* < 0.05; with *post hoc* Tukey's MCT (***e***) and the later phase from 300 to 600 s, one-way ANOVA: *F*_(3,243)_ = 5.04391, *p* < 0.05; with *post hoc* Tukey's MCT (***f***). ***g***, Bar graphs represent percentage pupariation of the indicated genotypes on an amino acid-deficient diet, one-way ANOVA: *F*_(3,12)_ = 120.0924, *p* < 0.05; with *post hoc* Tukey's MCT. Boxes and bars with the same alphabet represent statistically indistinguishable groups. ***h***, Schematic summarizing cholinergic activation (mAChR) and peptidergic modulation (FMRFaR, CCHa2R, and AstAR) of glutamatergic neurons required for pupariation on a PPD. GPCRs stimulate calcium release through the IP_3_R followed by SOCE in glutamatergic neurons. The intracellular calcium signaling regulates expression of genes encoding several ion channels as well as the mAChR. Activation of intracellular calcium signaling mechanisms and ion channels stimulates a complex calcium response across glutamatergic neurons upon amino acid withdrawal. The neuronal response is necessary for pupariation on the PPD. Exact *p* values are provided in Figure 1-1.

To test whether changes in ion channel expression also occur in the subset of glutamatergic neurons where Ca^2+^ transients are observed upon withdrawal of EAA, we isolated these neurons by FACS after genetic labeling with GFP (Fig. 7*g*,*h*). Because knockdown of *dStim* in glutamatergic neurons resulted in abrogation of calcium transients (Fig. 2*b*) and a pupariation defect on PDD (Fig. 2*a*), we tested the effect of *dStim* knockdown on the expression of specific ion channel genes identified from the RNA-seq. Approximately 0.5% of total cells in the CNS were identified as GFP-positive in the control sample where *UAS-eGFP* was driven with the *VGN6341-GAL4.* Knockdown of *dStim* had no effect on the number of GFP-positive cells (Fig. 7*i*). The sorted glutamatergic cells expressed *vGlut* (a marker for glutamatergic neurons) (Mahr and Aberle, 2006) at levels almost threefold higher than the housekeeping gene *rp49* (Fig. 7*j*). Significant reduction in *dStim* levels was observed from control normalized to the housekeeping gene *Act5c* upon knockdown of *dStim* (Fig. 7*k*), whereas levels of another housekeeping gene, β-*tubulin*, did not change between the two conditions (Table 2). These data indicate that sorting was specific to the *VGN6341-GAL4*-marked cells in the control and knockdown conditions. Importantly, mRNA levels of genes encoding mAChR (*mAChR-A*; Fig. 7*k*), and several voltage-gated ion channels specific for sodium (*NaCP60E*k), potassium (*Hk, eag*), and calcium (*cac, Ca*-α*1D*) were reduced upon knockdown of *dStim* (Fig. 7*k*). This observation was similar to gene expression changes observed in the whole larval CNS (Fig. 7*e*). Similar results were obtained when fold changes were calculated upon normalization to two other housekeeping genes: *rp49* and *tubulin* (Table 2). Moreover, knockdown of intracellular calcium signaling components in glutamatergic neurons also attenuated the calcium response to a depolarizing stimulus (Fig. 8*a*,*b*). These data support a role for SOCE in the maintenance of excitability in *VGN6341-GAL4*-marked glutamatergic neurons by regulating expression of ion channels.

To test whether reduction in ion channel gene expression is relevant for the calcium transients observed upon amino acid withdrawal, toxins specific to sodium, potassium, or calcium channels (Wu et al., 2008) were expressed with *VGN6341-GAL4*. Interestingly, such chronic inhibition of Na^+^, K^+^, or Ca^2+^ ion channels caused only a modest reduction in the percentage of responding cells and did not alter the nature of the transients in the initial phase after amino acid withdrawal (Fig. 8*c–e*). However, expression of every toxin altered maintenance of calcium transients in the latter phase from 300 to 600 s (Fig. 8*c*,*f*). Expression of these toxins also resulted in severe pupariation deficits on PDD (Fig. 8*g*). Together, these data identify regulation of ion channel gene expression changes as a mechanism by which the IP_3_R and resultant SOCE regulate neuronal function and plasticity in the larval CNS.

## Discussion

In this study, we have explored the neural mechanisms that control pupariation on a PPD. We find that presence of arginine in the environment serves as an important proxy for nutrition in *Drosophila* larvae. Loss of arginine is sensed by the amino acid transporter *slimfast* in peripheral sensory neurons that are cholinergic. This information is conveyed to glutamatergic interneurons in the VG, where it initiates calcium transients. Whereas arginine appears to be an important signal for sensing nutrient deprivation, other EAAs play smaller, and possibly additive roles for evoking calcium transients in glutamatergic interneurons and in pupariation. Neuropeptidergic modulation is equally necessary for the calcium transients seen in response to loss of amino acids. The calcium transients in turn stimulate neuropeptide secretion from the mNSCs, required for pupariation when the diet of mid-third instar larvae lacks protein. Both cholinergic and peptidergic inputs to the glutamatergic interneurons are dependent on receptors that stimulate intracellular calcium signaling through IP_3_R/SOCE. We have identified regulation of ion channel gene expression by IP_3_R/SOCE as a long-term mechanism for modulating neuronal function and plasticity of the glutamatergic interneurons (Fig. 8*h*).

### Sensing of arginine by cholinergic neurons through *slimfast*

Nutrient-sensing mechanisms have evolved in response to epochs of nutrient deprivation (Efeyan et al., 2015). Dietary arginine levels have also been shown to regulate insulin release (Liang et al., 2017). In newts, because of the cannibalistic nature of adult newts, arginine serves as a threat assessment for larvae (Ferrer and Zimmer, 2007a). Similar to newt larvae (Ferrer and Zimmer, 2007b), our findings suggest that *Drosophila* third instar larvae sense environmental levels of arginine. In *Drosophila* larvae, arginine presumably serves to estimate diet quality and availability. Biochemically, arginine is converted to ornithine and polyamines required for cell proliferation (Auvinen et al., 1992; Lange et al., 2014). This property of arginine may be significant for tissue remodeling during pupariation.

The ability of Class IV multidendritic sensory neurons in nociception and response to parasitoid wasp attacks is well established (Hwang et al., 2007; Zhong et al., 2010; Ohyama et al., 2015). However, their function in the context of nutrient deprivation is a recent finding (Jayakumar et al., 2016). In this study, we identify them as direct sensors of arginine. Recent reports have described how levels of dietary amino acids affecting their dendritic branching (Watanabe et al., 2017) and subsequently that nutrient-dependent changes in dendritic branching are distinct from their nociceptive function (Brown et al., 2017), thus strengthening the role of multidendritic sensory neurons in nutrient-sensing. We have explored the response to dietary deprivation of amino acids using a semi-intact preparation. Although this has been informative, systemic humoral responses would not have been measured, and these could well be relevant for pupariation on a PPD. With the existing improvements in imaging methods, it would be of interest to investigate the effects of nutrient deficiency on the decision to pupariate in freely behaving larvae as well.

Our data support expression of *slif* in multidendritic sensory neurons and functionally demonstrate that pupariation on PDD requires *slif* in *ppk-GAL4*-expressing neurons. In the fat body, *slif* is required for arginine transport (Colombani et al., 2003). A recent study on homologs of *slif*, however, revealed that it could function as a “transceptor,” a hybrid of a receptor as well as a transporter (Boudko et al., 2015). A transceptor role for Slimfast in sensory neurons is supported by our observation of loss of calcium transients in *ppk* neurons upon withdrawal of arginine in the absence of Slimfast (Fig. 6*e*). Interestingly, we also observed a significant decrease in the preference for arginine upon knockdown of *slif* in neurons marked by *ppk-GAL4*, suggesting that these larvae may be repelled by arginine. However, the regulation of acute diet preference through Slimfast needs to be investigated further.

### Neuronal regulation of pupariation

Response to starvation in *Drosophila* is thought to be primarily regulated by the fat body (Owusu-Ansah and Perrimon, 2014). Both the *Drosophila* brain and the fat body are tissues that exert metabolic control (Colombani et al., 2003; Hietakangas and Cohen, 2009; Bjordal et al., 2014). However, premature pupariation observed by optogenetic activation of VG glutamatergic interneurons (Fig. 1*g–i*) supports the idea that neuronal regulation described here might override other metabolic signals.

Calcium responses encoded by glutamatergic interneurons regulate ecdysteroid-synthesizing gene expression by stimulating peptide release from the mNSCs, and this release is essential for pupariation on PDD (Jayakumar et al., 2016). Therefore, we hypothesize that neuropeptide release from the mNSCs, which is regulated by the glutamatergic interneurons, regulates the expression of ecdysteroid-synthesizing genes. These cells produce various peptides, including Dilp2 whose release is affected by activity in glutamatergic interneurons of the VG (Jayakumar et al., 2016). The role for other neuropeptides, such as Dilp3, Dilp5, DSK, DH44, and SIFa (Brogiolo et al., 2001; Cabrero et al., 2002; Rulifson et al., 2002; Terhzaz et al., 2007; Park et al., 2008), produced by the mNSCs needs to be tested in pupariation during nutrient deprivation. Even though our data support the role of mNSC peptides, specifically Dilp2, in ecdysteroid synthesis and pupariation, they do not exclude the possibility that such regulation might also occur indirectly through modulation of the PTTH neurons (Nijhout and Williams, 1974; Mirth et al., 2005).

### Integration of metabolic state by glutamatergic interneurons

Interestingly, our data suggest that calcium transients from a fraction of neurons (amid glutamatergic interneurons of segments T3-A5 in the VG and marked by *VGN6341-GAL4*) encode the loss of dietary amino acids. The systemic response to loss of amino acids thus requires that the underlying cellular response reach a signaling threshold in a certain number of glutamatergic neurons from the potential responsive pool and not necessarily all the cells. Such a population code, for the integration of sensory information, is not unprecedented and has been shown in different contexts (Erickson, 2000; Carleton et al., 2010; Cohn et al., 2015; Tantirigama et al., 2017).

In addition to peptide release regulated by the glutamatergic interneurons, reduction in the environmental levels of amino acids elicits peptide release from other neuropeptidergic cells. Furthermore, the calcium transients in the glutamatergic neurons that encode loss of arginine are initiated by sensory inputs but also require inputs through multiple neuropeptide receptors. The knockdown of neuropeptide receptors altered the number of responding cells, and it is therefore likely that neuropeptidergic signals help propagate the calcium response initiated by cholinergic inputs. Whereas FMRFa and CCHa2 positively regulate transient propagation, AstA is a negative regulator of the response. Peptidergic modulation of neural circuits has been well studied in *Drosophila* and other invertebrates (Taghert and Nitabach, 2012).

CCHa2 is expressed predominantly in the fat body (Sano et al., 2015) as well as the brain and the midgut (Ren et al., 2015) of *Drosophila* larvae and conveys nutritional information about environmental proteins and carbohydrate levels in *Drosophila* larvae (Sano et al., 2015). The role of FMRFa in nutritional sensing is not understood, although FMRFa regulates glucose metabolism in snails (Röszer and Kiss-Tóth, 2014). AstA regulates feeding of *Drosophila* larvae depending on the available nutrient content in the environment (Hentze et al., 2015; J. Chen et al., 2016) and is known to negatively regulate feeding in adult flies (Hergarden et al., 2012).

The ability of different neuropeptides secreted from various internal tissues to drive antagonistic neuronal responses suggests that nutrient-sensing glutamatergic neurons are regulated differentially based on their expression of neuropeptide receptors (Fig. 8*h*). Knockdown of the putative hugin receptor PK-2 on *VGN6341-GAL4*-marked neurons also leads to pupariation deficits upon nutrient deprivation (Jayakumar et al., 2016). Peptidergic modulation of glutamatergic neurons likely allows for integration of external sensory cues (starvation in this case), with metabolic changes in the fat body, possibly the gut and the central brain. Thus, the glutamatergic interneurons are modulated by various neuropeptides in a state-dependent manner (starved vs nonstarved), further supporting complex neuromodulation for pupariation, similar to neuropeptidergic modulation of circuits that regulate ecdysis (Diao et al., 2017). Together, this integrated response at the appropriate developmental stage enables larvae to make the decision to continue development and pupariate in the absence of external nutrients. This finding supports the well-established concept of third instar larvae reaching “critical weight” when 50% of *Drosophila* larvae continue toward pupariation even under nutrient-deprived conditions (De Moed et al., 1999; Mirth et al., 2005).

### Modulation of neuronal response by IP_3_R through gene expression

The dependence of neuronal calcium transients on intracellular calcium signaling components as well as ion channels indicates that the two mechanisms work synergistically to generate this complex cellular response in a network of multiple neurons. This is a likely explanation for why the neuronal response is not sustained upon expression of ion channel toxins. IP_3_R and SOCE have been implicated in activity-dependent plasticity of mammalian neurons (Emptage et al., 2001; Rose and Konnerth, 2001), in the context of motor coordination (Fujii et al., 2000; Ichise et al., 2000; Hartmann et al., 2014) as well as for enabling LTP in hippocampal neurons (Fujii et al., 2000), synaptic plasticity in pyramidal neurons (Narayanan et al., 2010), and LTD in Purkinje neurons (Futatsugi et al., 1999; Hartmann et al., 2014). However, the mechanism(s) by which IP_3_R/SOCE alter neuronal function and plasticity are open to debate (Hartmann et al., 2011; Majewski and Kuznicki, 2015). It is possible that attenuation of IP_3_R/SOCE signaling reduces neuronal activity, which in turn affects the expression of ion channels encoding genes. Store-calcium release is known to increase the density of cation-nonspecific h channels in rodent hippocampus (Narayanan et al., 2010). Also, synaptic scaling is dependent on homeostatic plasticity modulated by changes in calcium levels (Ibata et al., 2008; Goold and Nicoll, 2010). Similar to our findings, IP_3_-mediated Ca^2+^ release and SOCE might affect ion channel gene expression in mammalian neurons and thus underlie aspects of neuronal plasticity in addition to other acute mechanisms by which IP_3_-mediated Ca^2+^ release and SOCE regulate ion channel function. Plasticity in the neural circuit(s) that respond to nutrient stress may be advantageous for adapting to the stress in adults as well and needs further investigation.
